# Factors influencing tuberculosis preventive practices among people living with HIV/AIDS enrolled in secondary health facilities in Lagos, Nigeria

**DOI:** 10.4314/ahs.v23i2.11

**Published:** 2023-06

**Authors:** Sunday Olakunle Olarewaju, Roseline Oluyemisi Akande, Charity Abu, Charles Adeyemo

**Affiliations:** 1 Department of Community Medicine, Osun State University, Osogbo, Nigeria; 2 Department of Community Medicine, LAUTECH, Ogbomoso, Oyo- State, Nigeria; 3 Ladoke Akintola University of Technology Post graduate School, Community Medicine Department, Osogbo Campus; 4 Department of Public Health, Faculty of Basic Medical Sciences, Adeleke University, Ede, Osun-State, Nigeria

**Keywords:** PLHIV, TB prevention, knowledge, attitude, practice

## Abstract

**Background information:**

Tuberculosis is the most potent opportunistic infection leading to deaths among patients with Human Immunodeficiency Virus (HIV). Therefore, knowledge, attitude and practices regarding tuberculosis preventive strategy among People Living with HIV/AIDS (PLWHA) is vital in minimizing the risk of developing TB infection which forms the objective of this study.

**Methodology:**

A descriptive cross-sectional study carried out using multi-stage sampling technique in selecting 606 respondents. Data was collected using a semi-structured questionnaire and analysed with SPSS version 23.

**Result:**

Of all the respondents, 245 (40.4%) were within 28-37 years, 386 (63.7%) were females and 219 (36.1%) had secondary education. Majority of the respondents 500 (82.5%), 423(69.8%) and 411(67.8%) had positive attitude, good practices and good knowledge respectively. Associated factors were age, marital status, religion, educational status, knowledge and attitude with tuberculosis practices. Respondents between 18-27 years were twice less likely to engage in good TB preventive practices (OR-0.44, 95% CI- 0.25-0.78, p = 0.004).

**Conclusion:**

Over half of respondents had good tuberculosis preventive practices, influenced by their socio-demographic characteristics, knowledge and attitude towards TB infection. Preventive efforts need to be strengthened among individuals between 18-27 years of age, non-Christians and those with lower educational status.

## Background

Tuberculosis is a disease of major public health concern and the most potent opportunistic infection as well as the leading cause of death among patients with Human Immunodeficiency Virus (HIV), particularly in sub-Saharan African and Asian countries, where it is highly prevalent 1-4. HIV increases the likelihood of reactivation and re-infection of Mycobacterium Tuberculosis (MTB) among people living with HIV/AIDS (PLWHA) 5. TB and HIV co-infection are increasing worldwide, most especially in developing countries. According to the World Health Organization (WHO), an estimated one-third of the 34 million HIV-infected people globally are infected with latent TB (6). Almost 20% of the global reported HIV-associated TB cases are found in South Africa with a co-infection rate of 73% while in Sub-Saharan Africa, the co-infection rate is also as high as 50 - 70% 7,8 Between 2000 and 2016, TB treatment averted an estimated 44 million deaths among HIV-negative people while among HIV-positive people, TB treatment supported by ART averted an additional 9 million deaths 9.

The WHO African region accounted for 25% of the total number of incident cases (i.e., TB-HIV-negative and TB-HIV infection) globally, where Nigeria accounted for 8% or 407 cases per 100,000 population in 2016, up from 322 cases per 100,000 population in 2015. These estimates may be lower than the actual number of TB cases in Nigeria because only less than a quarter of TB cases (15%) were notified in 2015, therefore, it is not surprising that Nigeria still remains one of the 30 high burden countries for tuberculosis (TB), TB/HIV, and Multidrug-Resistant TB (MDR-TB) globally 10,11.

Consequently, the dual epidemics of TB and HIV have raised the need for the implementation of collaborative TB/HIV programmes, in order to reduce the burden of TB among PLWHA and the burden of HIV among people with TB using the 3Is (Three “Is” strategy) by WHO 12. The fulcrum of this strategy includes intensified case finding for TB (ICF), isoniazid preventive therapy for TB (IPT) and TB Infection Control (IC)^13^. Despite the recommendations by World Health Organization and the considerable benefits of prompt TB screening among HIV/AIDS patients, HIV programmes have been slow to implement these TB-reducing services, resulting in missed opportunities to prevent many unnecessary cases of TB and related deaths 13

TB screening among PLHIV is faced with a number of challenges, which includes a lack of knowledge amongst people living with HIV about tuberculosis, persistently high transmission rates and delay in health-seeking behaviour 14. A study done among HIV clients in Minna, North-central Nigeria found that 52.6% of them had never been screened for TB infection, indicating the need for more efforts to screen all PLWHA for TB 2. Delayed diagnosis of TB among PLWHA may be associated with increased mortality and morbidity, thus an intensive screening for latent/active TB is vital to decrease the incidence of TB at every clinical encounter. If the factors affecting TB preventive services are not adequately addressed particularly among PLWHA, it could undermine the giant strides made globally in rapidly expanding HIV care and treatment. This also underscores an urgent need to build better TB prevention in resource- limited settings, such as Nigeria.

However, in order to identify factors that could contribute to low rates of TB screening among HIV positive clients, it is imperative to assess the practices of HIV patients regarding TB preventive strategies. The results of such an assessment will be helpful to policy makers and healthcare providers in developing strategies tailored towards curtailing high co-infection rate and thereby improve TB and HIV treatment outcomes. Therefore, this study was conducted to assess the factors influencing tuberculosis preventive practices among people living with HIV/AIDS enrolled in secondary health facilities in Lagos, Nigeria.

## Materials and Methods

### Study Location

The study was conducted in Lagos State which is located in South West region in Nigeria. Lagos State is the commercial nerve center of Nigeria, although it is the smallest state in the country in terms of land mass. The state hosts about 11.4% of the Nigerian population and is divided into 20 Local government areas (LGAs) and 37 local council development areas 15. DOTS management of TB commenced in 2003 in collaboration with the WHO and the United States Agency for International Development (USAID) in Lagos State. At inception, DOTS activities were implemented in 3 of the 20 LGAs, but within 3 years all the LGAs in the state had at least one health facility offering TB treatment services. At the end of 2015, there were (103 private and 210 public health facilities) offering DOTS TB services in the State. 15

Lagos has a deep harbor port with a well-established commercial status which makes it attractive for thousands of job seekers from other parts of the country. This has led to overcrowding in poorly ventilated accommodations, high unemployment, increased slum dwellers and poverty, creating favourable conditions for the high prevalence of diseases such as TB, and HIV. In 2016, Lagos reported 8,128 TB cases of all forms of TB 16. Regarding HIV programs, Lagos state reported that 1,537 new cases were enrolled in HIV care. Out of these 522 (43%) were co-infected with TB, 802 (79%) were started on isoniazid preventive therapy IPT 16.

### Study Design and Population

This was a descriptive cross-sectional study, conducted among HIV positive clients aged 18 years and older enrolled into treatment in secondary health care facilities for more than 6 months before the commencement of the study. HIV clients that were pregnant and had psychiatric disorders were excluded from the study.

### Sample Size Determination and Sampling Technique

The minimum sample size of 322 was calculated using the Leslie Fischer's method (17), at a confidence level of 95%. Adding 10% non-response rate, this was increased to 354 but a total of 606 questionnaires were administered.

n= Z2pq/d2 where n is the sample size, Z – standard normal deviate (1.96), P is the proportion of HIV client's uptake of IPT from previous study – 30% (18), q is 1-p, (0.7) while d is the error margin 0.05.

A multi-staged sampling technique with 3- level approach was used to select participants for the study.

### Stage one: Selection of local government areas

Two LGAs each were selected from each of the 3 senatorial zones of Lagos state giving a total of 6 LGAs. Using simple random sampling by balloting, Shomolu and Kosofe LGAs were selected from Lagos East senatorial zone, Agege and Ajeromi LGAs were selected from Lagos-West zone and Surulere and Apapa LGAs were selected from Lagos-Central zone.

### Stage two: Selection of health facilities

Six Secondary Health facilities were selected out of all comprehensive list of secondary health facilities collated in selected two LGAs offering comprehensive HIV treatment and care services using random sampling by balloting.

### Stage three: Selection of study participants

Selection of people living with HIV on HAART for at least 6 months were done using systematic random sampling based on proportional allocation across the six selected secondary health facilities used until desired sample size was obtained.

### Research instrument and data collection methods

A structured interviewer-administered questionnaire was used as the study instrument. This was designed to seek information about the HIV positive patients' socio-demographic characteristics, knowledge about the TB infection prevention and control strategies, questions on the attitude and practices of patients concerning TB prevention strategies as well as their expectations towards accessing TB screening services. Research assistants comprising of HIV field workers supporting health facilities in the delivery of community HIV screening and who were familiar with the HIV patients were engaged and trained before data collection.

### Validation and pre-test of the instrument

The validity and reliability of the questionnaire were done before the final collection of data. Two Nigerian experts in the field of Epidemiology in a Nigerian university evaluated the extent to which the variables in the questionnaires were relevant to the objectives of the study. Thereafter, the questionnaire was pretested among eligible HIV positive patients attending health facilities in Lagos state General Hospital, which was not among the selected facilities for the study. This helped to know whether the questionnaire assessed what it intended to measure and whether the language and organization were appropriate to address the objectives of the study. The responses provided also helped to address any form of ambiguity in the questionnaire as well as modified questions or response categories where necessary. The Cronbach's alpha coefficient of the questionnaire was 0.82, indicating an acceptable internal consistency.

### Measurement of outcome variables

The question about knowledge and attitude were scored. For questions whose responses were either yes or no, a correct answer was scored 1 and a wrong answer was scored 0. For questions with three responses, (Yes, No and I don't know), correct response was scored 2, don't know scored 1 while wrong response scored 0. The attitude of the respondents towards tuberculosis infection were assessed using five items on a five-point Likert scale ranging from strongly disagree (1), disagree (2), indifferent (3), agree (4), to strongly agree (5). Thereafter, the responses were coalesced into three categories; agree, not sure and disagree. The mean score of the maximum score for knowledge and attitude respectively were calculated. The respondents who scored below the mean were regarded as having poor knowledge or negative attitude while those who scored up to or above mean were regarded as having good knowledge or positive attitude.

### Data Analysis

Questionnaires were sorted out to check for errors and omissions at the end of collection of data between May and June, 2021. Thereafter, data were entered into the computer and analysed using Statistical Package for Social Sciences (SPSS) version 23. Frequency distribution tables, charts and graphs were generated from variables while cross tabulation and test statistics was done where applicable. Chi square was used to compare rates, ratios and proportions while fishers' exact test was used when cells have expected values less than 5. Student T test was used to determine the association between the continuous variables. A binary logistic regression analysis was used to identify the determinants of TB preventive practices among respondents, using the demographic variables as independent variables and good preventive practices as the outcome variable. All the significant variables during the bivariate analysis were imputed into the logistic model. Adjusted odds ratio (AOR) and 95% confidence interval were also obtained to identify the determinants of TB preventive practices among the respondents. The level of significance was set at p-value less than 0.05.

## Results

Table 1 shows that less than half of the respondents according to the socio-demographic status 245 (40.4%) were within 28-37 years of age, 386 (63.7%) of them were females and 219 (36.1%) had secondary education. Out of these, 391(64.5%) were Christians, 369 (60.9%) of Yoruba ethnic descent, 381 (62.9%) were married while 354 (58.4%) were skilled respectively.

**Table 1:** Socio-demographic characteristics of respondents

Variable	Frequency (n=606)	Percentage (%)
**Age in years**		
18-27 years	149	24.6
28-37 years	245	40.4
Greater than 38 years	212	35.0
**Gender**		
Male	220	36.3
Female	386	63.7
**Highest level of education**		
No education	28	4.6
Primary education	140	23.1
Secondary education	219	36.1
Tertiary education	219	36.1
**Religion**		
Christian	391	64.5
Muslim	206	34.0
Traditional	9	1.5
**Ethnicity**		
Yoruba	369	60.9
Hausa	59	9.7
Igbo	141	23.3
Others	37	6.1
**Marital status**		
Single	169	27.9
Married	381	62.9
Divorced	10	1.7
Separated	17	2.8
Others	29	4.8
**Occupation**		
Professional	118	19.5
Skilled	354	58.4
Unskilled	69	11.4
Housewife	15	2.5
Unemployed	27	4.5
Others	23	3.8

According to table 2, almost all the respondents, 584(96.4%) were aware of tuberculosis while 412 (68%) knew about bacteria as a causative agent, 400(66%) knew HIV/AIDS, smoking as risk factors while 600 (99%) knew cough, weight loss and night sweat as symptoms. Concerning organs affected with tuberculosis, 538(88.8%), 18(3.0%), 18(3.0%) mentioned lungs, kidney and liver respectively. Also, 551(90.9%) knew tuberculosis can be transmitted as airborne infection, 568 (93.7) knew HIV infected individuals can have tuberculosis while 591(97.5%) knew tuberculosis is curable infection among HIV positive individuals. Concerning knowledge on diagnosis, majority i.e., 447(73.8%) knew sputum test while only 15 (2.5%) mentioned gene expert machine. Regarding knowledge on treatment, only 15(2.5%) knew four drugs (rifampicin, isoniazid, Pyrazinamide and ethambutol) used in the treatment of tuberculosis during intensive phase while 90 (14.9%) mentioned rifampicin and isoniazid as drugs used during the intensive phase. Regarding knowledge on preventive measures, majority i.e., 469(77.4%) and 516 (85.1%) knew taking isoniazid tablet and constant clinic visitation respectively as preventive measures while 322(53.1%) mentioned burying sputum in the ground.

**Table 2:** Knowledge on TB infection and its management among respondents

Variable	Frequency (n=606)	Percentage (%)
**Awareness of Tuberculosis**	584	96.4
Bacteria as cause of Tuberculosis	412	68.0
HIV, Smoking, PLHIV as risk factors	400	66.0
Cough and weight loss night sweat as symptoms	600	99.0
Lung as organ affected by Tuberculosis	538	88.8
Kidney as organ affected by Tuberculosis	18	3.0
liver as organ affected by Tuberculosis	18	3.0
Airborne route as mode of transmission of tuberculosis	551	90.9
HIV individual can have Tuberculosis	568	93.7
Tuberculosis is curable infection	591	97.5
Sputum test can diagnose Tuberculosis	447	73.8
Blood test can diagnose Tuberculosis	74	12.2
Chest X-ray can diagnose tuberculosis	46	7.6
AFB, Gene expert can diagnose tuberculosis	15	2.5
**Knowledge on drugs used in treatment**		
Isoniazid	215	35.5
Ethambutol and Isoniazid	17	2.8
Pyrazinamide	11	1.8
Rifampicin and Isoniazid	90	14.9
Rifampicin, isoniazid, Pyrazinamide and Ethambutol	15	2.5
Cotrimoxazole	9	1.5
**Knowledge on Preventive measures**		
Opening one's mouth while coughing at home	63	10.4
Constant clinic visits to ask of symptoms of tuberculosis	516	85.1
Correct taking of isoniazid tablet among HIV positive clients	469	77.4
Not shaking hands with individuals that is coughing or confirmed to have tuberculosis	82	13.5
Burying sputum in the ground among individuals that are coughing	322	53.1
Having all children less than 5 years and household members doing TB test will prevent TB infection within the community	335	55.3
Not drinking water with someone confirmed with tuberculosis infection	257	42.4
Not using the same toilet with someone confirmed with tuberculosis infection	55	9.1

*Multiple responses allowed

In terms of attitude, 552 (91.1%), 44(7.3%), 9(1.5%), 389(64,2%) and 485 (80%) of the respondents agreed on clinical screening of household members and subsequent laboratory testing, use of traditional herbs among TB tested positive individuals, mandatory TB testing among HIV positive clients and TB treatment as protective measure as preventive attitudes respectively. (Table 3)

**Table 3:** Attitude of respondents towards Tuberculosis infection

Variables (n=606)	Agree	Disagree	Not sure
All HIV clients' partner and their household members should be screen clinically and do laboratory test for TB.	552(91.1%)	43(7.1%)	11(1.8%)
All positive TB test result must use traditional herds as a treatment measure.	44(7.3%)	538(88.8%)	24(4%)
Testing positive for TB among HIV positive is a death sentence	9(1.5%)	563(92.9%)	34(5.6%)
TB testing among HIV positive clients must be mandatory	389(64.2%)	131(21.6%)	86(14.2%)
TB treatment among HIV Positive TB client is a protective measure	485(80%)	18(3%)	103(17%)

Regarding TB preventive practices among the study participants, 540(89.1%) cover their mouth while coughing, 100 (16.5%) bury sputum and cover with sand, 506 (83.5%) did not observe cough etiquettes, 400(66.0%) had taken isoniazid preventive therapy while 366 (60.4%) had household members ever screened for tuberculosis. (Table 4)

**Table 4:** Tuberculosis Preventive Practices among respondents

Variable	Frequency (n=606)	Percentage (%)
Covering of mouth while coughing	540	89.1
Bury sputum and cover with sand	100	16.5
Did not observe cough etiquette	506	83.5
Taking isoniazid Preventive therapy	400	66.0
Had never screened household members for TB	366	60.4

*Multiple responses allowed

Out of 606 respondents, 500 (82.5%), 423(69.8%) and 411(67.8%) had positive attitude, good practices and good knowledge of preventive practices towards tuberculosis infection respectively (Table 5).

**Table 5:** Categorized knowledge, Attitude and TB Preventive Practices towards Tuberculosis infection among respondents

Variable	Frequency (n=606)	Percentages (%)
Positive attitude	500	82.5
Negative attitude	106	17.5
Good Practice	423	69.8
Poor practice	183	30.2
Good knowledge	411	67.8
Poor knowledge	195	32.2

The result of the bivariate analysis, showed that there is an association between age in categories (χ^2^- 9.93, P- 0.002), marital status (χ^2^- 15.62, P-0.00), religion (χ^2^– 30.93, P = 0.00), educational status (χ^2^ – 37.58, P-0.00), categorized knowledge (χ^2^ – 20.86, P = 0.00), categorized attitude (χ^2^– 9.15, P = 0.002) with categorized Tuberculosis practices (Table 6).

**Table 6:** Bivariate and logistic regression of selected variables and categorized Tuberculosis Preventive Practices

Bi-variate analysis	TB Preventive Practices		X^2^	P
	Good	Poor		
Age in categories			9.925	*0.002
18-27 years	92(61.7%)	57(38.3%)		
Greater than 28 years	331(72.4%)	126(27.6%)		
Marital Status			15.62	*< 0.01
Never married	138(81.7%)	31(18.3%)		
Ever married	285(65.2%)	152(34.8%)		
Ethnicity			3.67	0.06
Yoruba	247(66.9%)	122(33.1%)		
Non-yoruba	176(74.3%)	61(25.7%)		
Religion			30.93	*<0.01
Christian	303(77.5%)	88(22.5%)		
Non-christians	120(55.8%)	95(44.3%)		
Education status			37.58	*<0.01
Not educated	5(17.9%)	23(82.1%)		
Educated	418(72.3%)	160(27.7%)		
Categorized knowledge				
Good knowledge	311(75.7%)	100(24.3%)	20.86	*< 0.01
Poor Knowledge	112(57.4%)	83(42.6%)		
Categorized attitude			9.15	*0.002
Good attitude	362(72.4%)	138(27.6%)		
Poor	61(57.5%)	45(42.5%)		
attitude				

Logistics regression (Good Tuberculosis Preventive Practices)								
	OR	95%CI		P value	AOR	95%CI		P value
		Lower	Upper			Lower	Upper	
Age (Ref - >28 years)	0.44	0.25	0.78	0.004	2.25	1.29	3.94	*<0.01
Marital Status (Ref =Never married)	3.36	1.89	5.94	0.00	3.36	1.89	5.94	*<0.01
Religion (Reference = non-Christians)	1.78	1.19	2.65	0.005	0.56	0.38	0.84	*0.01
Educational status (Reference = Education)	0.14	0.05	0.38	0.000	7.33	2.63	20.39	*<0.01
Categorized knowledge (Reference = Good knowledge)	1.46	0.94	2.24	0.089	1.46	0.94	2.24	0.09
Categorized attitude (Reference =good attitude)	1.47	0.89	2.42	0.132	1.47	0.89	2.42	0.13

* Statistically significant

At multivariate levels, the logistic regression showed that, individuals between 18-27 years were twice less likely to indulge in good TB preventive practices (OR- 0.44, CI-0.25-0.78, P = 0.004, AOR = 2.25, CI- 1.29 – 3.94, p = <0.01). Also, ever married individuals are 3 times more likely to indulge in good practices compared to those that never married (OR- 3.36, CI 1.89-5.94, P = 0.00, AOR = 3.36, CI- 1.89 - 5.94, p = <0.01). In addition, Christians were twice likely to engage in good Tuberculosis preventive practices compared to non-Christians (OR- 1.78, CI-1.19-2.65, P = 0.00, AOR = 0.56, CI- 0.38 – 0.84, p = 0.01). In terms of education, non-educated respondents were 7times less likely to engage in good Tuberculosis preventive practices compared to those that are educated (OR- 0.14, CI- 0.05-0.38, P = 0.00, AOR = 7.33 CI- 2.63 - 20.39, p = <0.01) (Table 6).

## Discussion

This study offers information on the knowledge, self-reported attitudes and preventive practices towards tuberculosis. The study found a significant gap in preventive practices towards TB infection control. This study also showed that the knowledge about TB was high among the respondents. This is consistent with the findings from a previous study in Minna, North-Central Nigeria, which reported a high knowledge of TB (73.5%) among PLHIV receiving treatment and care at the ART clinic 2. However, this finding is in contrast with the results of the Federal Ministry of Health [Nigeria] TB KAP (Knowledge, Attitude and Practice) Survey of the general population in Lagos state in 2012, which indicated that lower proportion of people were aware of the signs and symptoms of TB in the general population 19. It is believed that knowledge about TB among PLWHA is an important determinant of health- seeking behaviour as well as adherence to preventive measures and treatment 20.

Regarding preventive therapy uptake, more than half of the participants who were eligible have received IPT in the past. For those who were co-infected with TB and on anti-TB treatment, nearly two-thirds have not had their household members screened for TB. In this study, uptake of IPT was low among HIV positive participants eligible for this therapy compared to the national average of 74% 21.

The reason for this is not clearly understood. IPT is offered to all HIV positive patients who do not have TB, with a national target of 100 percent 16.

The reason for the low uptake in Lagos state could be due to attitudinal problems, as Lagos state has a well-structured public health system with the presence of a dominant private health sector players compared to other cities in Nigeria. The findings also showed that HIV positive patients are hesitant to take the TB test because of the fear of being stigmatised. This finding is in line with findings of other studies in Western Cape 22. It can, therefore, be argued that there is a need to enhance awareness and education on the benefits of TB screening and to offer continuous counselling on TB screening to HIV patients on subsequent visits to healthcare facilities. The findings of this study also revealed that two-thirds of respondents were females. This result contradicts reports from previous studies in Plateau State, North-Central and Lagos State, South-western Nigeria, which found that 63.2% and 75.4% of their study participants respectively were males 23,24. The differences in this study may suggest that females have better health seeking behaviour, increased level of awareness and education on TB.

With regards to age, majority of the respondents fell into the active age group of 28 to 37 years. The National Agency for Control of HIV/AIDS Annual Report 2011/2012 reported similar results in Lagos State where majority of TB patients were in the 25 to 44 years' age group 16.

Concerning education, the study findings indicate that only 4.6% of the respondents never attended school while the rest of the respondents attained a certain form of schooling with over one-third attaining tertiary education. This indicates a high literacy level in the State.

The findings also showed that the majority of the respondents were employed. However, most of them are skilled workers, while less than a third of the respondents are professionals. It is generally accepted that people with some forms of income are less likely to suffer from conditions that can lead to exposure to TB infection, since TB largely affects the disadvantaged individuals 25. However, with the emergence of HIV/AIDS, income has become a contributing factor to risky sexual behaviour which can lead to HIV infection transmission and subsequently to TB infection. The results of this study are similar to those reported by Médecins Sans Frontiers in Thailand who also found that the majority of respondents were employed 22.

The findings showed that majority of the respondents had a high level of knowledge about TB. This suggest that the level of knowledge on prevention TB and HIV infection is high among the majority of TB clients in Lagos State. It can be argued that this high level of awareness of prevention is due to ongoing health education campaigns on TB and HIV prevention at state and national levels. The result is similar but higher than the findings of a study in Ethiopia on knowledge, attitude and preventive practice towards tuberculosis among clients using public health facilities. The study reported that 54% of their respondents had a good knowledge about TB and its prevention and treatment 26.

Similarly, Getahun, Gunneberg, Granich & Nunn survey on employee knowledge, attitude and practices relating to TB and HIV/AIDS at a mining company in Namibia, also found that majority of the respondents had average knowledge of TB and HIV transmission and prevention 27. The findings reveal that there is a significant relationship between education and the level of knowledge on TB prevention. This suggests that the level of a respondent's education influences the level of knowledge on TB prevention. These findings are consistent with the findings by Harries, Zachariah & Lawn which found that co-infection of TB with HIV is mainly prevalent in countries with low levels of education 28. This may be explained by the fact that education enables one to access and process information and knowledge even in the area of TB and HIV prevention.

This study found that the attitude of majority of the respondents towards TB prevention services is positive. The results may be explained by the fact that the respondents with HIV positive status were aware of the benefits of accessing TB screening and testing services. A South African study conducted by Wood et al similarly found that 75% of respondents with HIV positive status had positive feelings towards testing services and were willing to take TB test in order to know their TB status 29. In this study, differences in gender of respondents did influence their attitude towards TB prevention. Female respondents are more likely to exhibit good practice towards TB prevention and screening than the male respondents.

Majority of the respondents had a positive attitude towards TB prevention programs. The positive attitude of HIV positive patients in Lagos State may possibly be due to the fact that these patients have been exposed to prevention and care programs through counselling and testing. The patients, therefore, understood the benefits of the programs. The results reveal a significant association between good practice towards TB prevention and care with education and occupation. Abebe and Mitikie in their study conducted in Ethiopia also found high levels of willingness to test among more educated people 30. This suggests that education and occupation influence the willingness to be tested for TB and practice good TB prevention measures.

## Conclusion

The study found that more than half of the respondents had good tuberculosis preventive practices. This finding was influenced by the age, educational status, religion, ethnicity, marital status, knowledge and attitude towards tuberculosis infection. It is therefore recommended that preventive efforts be strengthened among individuals between 18-27 years of age, non-Christians as well as those with lower educational status. Targeted health education for HIV/AIDS patients with low level of education may also significantly improve their knowledge about TB preventive practices.

## Study Limitation

This was a descriptive cross-sectional study design and social desirability bias could have been introduced because of the use of the interviewer-administered type of data collection tool. However, the research assistants were well trained to minimize this bias.

## Data Availability

Data can be made available upon request by external researchers and the corresponding author should be contacted to do so.

## Right of decline/withdraw from study

Respondents were told that participation is voluntary and they would not suffer any consequences if they chose not to participate in the study.

## Confidentiality of data

All information gathered were kept confidentially. Participants were identified using serial numbers; names of respondents were not requested.
